# 5-Fluoroindole Reduces the Bacterial Burden in a Murine Model of *Mycobacterium tuberculosis* Infection

**DOI:** 10.1021/acsomega.4c03981

**Published:** 2024-07-16

**Authors:** Christiano E. Neves, Josiane D. Paz, Bruno L. Abbadi, Raoní S. Rambo, Alexia M. Czeczot, Nathalia D. M. Sperotto, Adilio S. Dadda, Rodrigo B. M. Silva, Marcia A. Perelló, Guilherme A. Gonçalves, Laura C. González, Cristiano V. Bizarro, Pablo Machado, Luiz A. Basso

**Affiliations:** † Instituto Nacional de Ciência e Tecnologia em Tuberculose, Centro de Pesquisas em Biologia Molecular e Funcional, Pontifícia Universidade Católica do Rio Grande do Sul, 90616-900 Porto Alegre, Rio Grande do Sul, Brazil; ‡ Programa de Pós-Graduação em Biologia Celular e Molecular, Pontifícia Universidade Católica do Rio Grande do Sul, 90616-900 Porto Alegre, Rio Grande do Sul, Brazil; § Programa de Pós-Graduação em Medicina e Ciências da Saúde, Pontifícia Universidade Católica do Rio Grande do Sul, 90616-900 Porto Alegre, Rio Grande do Sul, Brazil

## Abstract

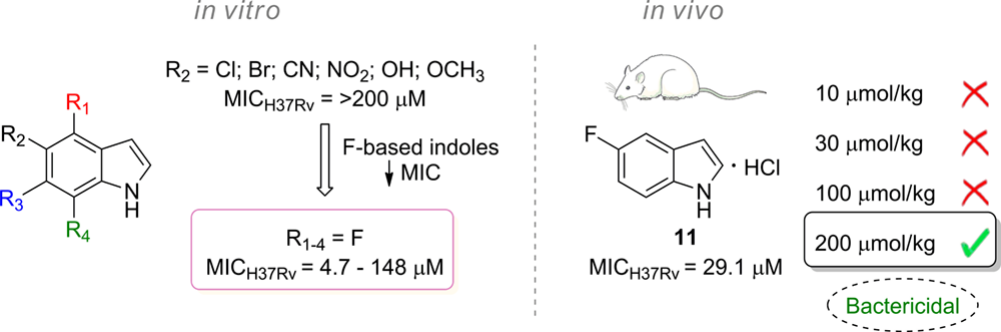

Tuberculosis is a disease caused by a single pathogen that leads to a death toll estimated to be more than a million per year. *Mycobacterium tuberculosis* (Mtb), which affects mainly the lungs, spreads by airborne transmission when infectious respiratory particles from an infected human enter the respiratory tract of another person. Despite diagnosis and treatment being well established, the rise of cases of patients infected with Mtb strains with multidrug resistance to the antibiotics used in the regimen against the disease is alarming. Indole used as a core molecule has been described as a promising structure to treat several diseases. 5-Fluoroindole (5-FI) compound, evaluated in the free base and in the hydrochloride (5-FI.HCl) forms, inhibited the growth of pan-sensitive Mtb H37Rv strain in the same range (4.7–29.1 μM) of clinical isolates that have resistance to at least two first-line drugs. Although 5-FI showed no cytotoxicity in Vero and HepG2 cells, high permeability (2.4.10^–6^ cm/s) in the PAMPA assay, and high metabolic stability (Cl_int_ 9.0 mL/min/kg) in rat liver microsomes, limited solubility at plasmatic and intestinal pH values prompted formation and employment of its salt form (5-FI.HCl). Although the 5-FI.HCl compound showed increased solubility at pH values of 7.4 and 9.1 and increased stability in aqueous solutions, data for intrinsic clearance (Cl_int_ = 48 mL/min/kg) and a half-life (*t*_1/2_ = 12 min) showed decreased metabolic stability. As 5-FI.HCl showed both good absorption and ability to reach the systemic circulation of animals without the need to use vehicles containing cosolvents or surfactants, it was chosen to evaluate its effectiveness in the model of tuberculosis in mice. The in vivo results showed the concentration of the compound in plasma increasing within 30 min in the systemic circulation and the capacity of reducing the Mtb burden in the lungs at the concentration of 200 μmol/kg after 21 days of infection, with no toxicity in mice.

## Introduction

Finding new antibiotics is a matter of increasing public health concern as drug-resistant bacteria spread around the world. However, this task is notoriously difficult as besides the challenge of developing safe and effective drugs, new antibiotics must be used sparingly to prevent resistance, thereby slowing sales. Emerging viral and bacterial infections pose a significant threat to global public health.^1^ Among the bacterial infectious diseases, tuberculosis (TB) is one of the most prevalent. Caused mainly by *Mycobacterium tuberculosis* (Mtb), airborne transmission of TB occurs when infectious respiratory particles expelled into the air by an infected human being (the source) enter the respiratory tract of another person. Despite adequate diagnosis and treatment being available, TB remains one of the leading causes of death worldwide. In 2021, TB affected an estimated 10.6 million people globally, with 1.4 million deaths among HIV-negative individuals, according to the World Health Organization.^2,3^ Among the HIV-positive population, an additional of 178,000 deaths were attributed to TB. Of concern is the emergence of drug-resistant TB strains, which is a major challenge to effective treatment as the first-line drug regimen is no longer effective.^2,3^ The prolonged treatment and lack of adherence to the regimen contribute to noncompliant outcomes, exerting selective pressure on the Mtb population leading to the emergence of rifampicin-resistant, multidrug-resistant, and extensively resistant strains of *M. tuberculosis*.^2,3^ The path to develop a drug to treat TB is long, and due to the characteristic hurdles of Mtb, many candidate compounds have been tested with limited success.^4^ As part of an ongoing research program, our research group is interested in identifying compounds capable of inhibiting drug-susceptible and drug-resistant Mtb strains. Among the molecules that have been evaluated, quinoline derivatives^5−7^ and their simplified analogues, including indoles,^8^ have shown encouraging results. In particular, indole-containing compounds have shown antimycobacterial activity using a diverse range of mechanisms of action, such as inhibiting cell wall synthesis, mycolic acid synthesis, and biosynthesis of essential amino acids.^9^ These molecules have also been shown to inhibit RNA synthesis, ATP synthase, DNA topoisomerase, and DNA gyrase enzymes.^9^

The tryptophan biosynthetic pathway has been described to be essential to the bacillus virulence.^10−12^ Mutations in the *trpD* gene have been shown to result in auxotrophic strains for tryptophan, which are avirulent and eliminated upon inoculation in both immunocompetent and immunodeficient mice.^10^ These findings indicate that the *trpD* gene is essential for Mtb virulence and that changes in this gene could lead to the generation of attenuated strains with potential use as vaccine candidates.^10^ Deletions of *trpE* and *trpA* genes have resulted in auxotrophic strains for tryptophan that lack virulence when inoculated in mice.^11,12^ Accordingly, these data suggest that the tryptophan pathway is essential for Mtb pathogenesis and virulence.

**Scheme 1:**
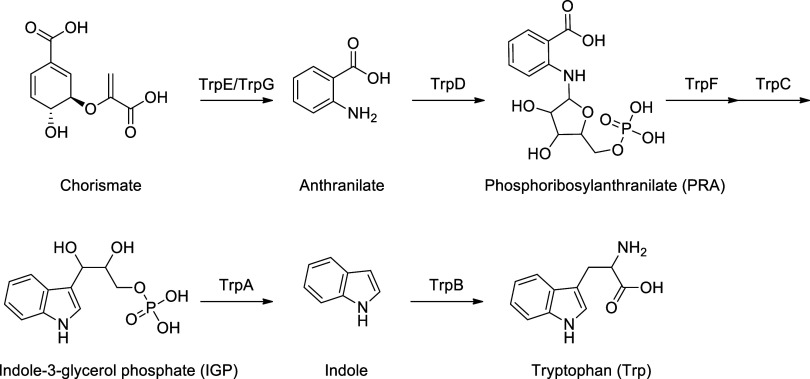
*Mycobacterium tuberculosis* Tryptophan Biosynthesis

The tryptophan biosynthesis process involves six enzymatic conversions (as depicted in Scheme 1). The first step is the synthesis of anthranilate from chorismate catalyzed by anthranilate synthase (TrpE/TrpG), a heterodimeric enzyme. This step is followed by condensation of anthranilate with 5′-phosphoribosyl-1′-pyrophosphate (PRPP) to produce phosphoribosyl-anthranilate (PRA) catalyzed by anthranilate phosphoribosyltransferase (TrpD) enzyme. The conversion of PRA into indole-3-glycerolphosphate (IGP) is carried out by the TrpF (PRA isomerase) and TrpC (indole-3-glycerol phosphate synthase) enzymes. Finally, the heterotetrameric tryptophan synthase complex (TrpAB) catalyzes the last two steps: the α subunit cleaves the indole ring from the glycerol phosphate backbone of IGP to form indole and glyceraldehyde-3-phosphate (G3P), and the β subunit condenses indole with l-serine (l-Ser) to form L-tryptophan (l-Trp).

**Figure 1:**
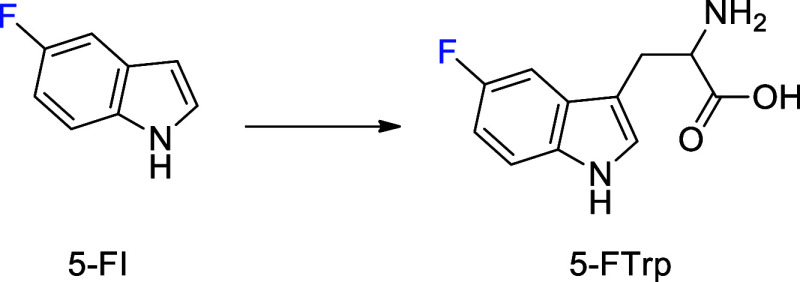
Formation of 5-FTrp from 5-FI.

As l-Trp is both the rarest amino acid in most proteins and most energetically demanding to produce, its biosynthesis is highly regulated at the transcriptional level and through allosteric control of enzyme activity.^13^ In mycobacteria, tryptophan biosynthesis is not transcriptionally controlled,^14^ and expression of tryptophan biosynthetic genes is constitutive even in the presence of exogenous tryptophan.^15^ These data suggest that mycobacteria synthesize amino acids irrespective of environmental availability. However, tryptophan restricts its biosynthetic pathway in mycobacteria through feedback inhibition of chorismate and anthranilate synthesis.^12,16^ Various chemical structures have been identified as inhibitors of the synthetic steps involved in tryptophan biosynthesis. For instance, the TrpAB protein complex is inhibited by azetins, sulfolanes, and indoline-5-sulfonamides.^12,17,18^ Interestingly, indole propionic acid, which is a metabolite produced by several species of bacteria found in the normal gut microbiota (including species of *Clostridium* and *Peptostreptococcus*), is an allosteric inhibitor of *M. tuberculosis* TrpE.^19^ Benzoates and dinitroaniline act on the tryptophan biosynthesis pathway by competitively inhibiting TrpD.^20^ Fluoroanthranilates, which are enzymatic substrates in the pathway, inhibit the growth of Mtb cultures and have been described as responsible for the inhibition of the nonvirulent strain of *M. tuberculosis* mc^2^ 6230.^21^ The Mtb mc^2^ 6230 strain (ΔRD1 ΔpanCD mutant) has a deletion of the RD1 locus, inherent to BCG strains, and an additional mutation (ΔpanCD) that makes the mycobacteria strictly auxotrophic for pantothenate.^22^ Attenuation obtained with mutations in the Mtb mc^2^ 6230 strain has allowed for its exploration as a potential vaccine candidate. Recently, the antimycobacterial activity of indole-4-carboxamides has been correlated with the metabolization of these structures and the concomitant release of 4-aminoindole, which is incorporated into the tryptophan biosynthesis pathway, forming the 4-aminotryptophan derivative.^23^ This molecule (4-aminotryptophan) has been identified as responsible for the inhibitory activity on Mtb growth. Therefore, we propose the use of 5-fluoroindole (5-FI) and its derivatives as potential inhibitors of Mtb growth and promising drug candidates for the treatment of TB. Our premise is based on the observed ability of fluoroanthranilates to produce fluorinated tryptophans that inhibit the growth of the *M. tuberculosis* mc^2^ 6230 strain.^21^ Accordingly, the use of 5-FI could facilitate the formation of 5-fluorotryptophan (5-FTrp) (Figure 1) at a reduced metabolic and energy cost as it would be closer to the final product of the biosynthetic pathway.

## Results and Discussion

The ability of 5-FI to inhibit the growth of the H37Rv strain of Mtb was evaluated by resazurin reduction microplate assay (REMA) using the first-line drug isoniazid (INH) as a positive control. Derivatives of 5-FI containing substituents at the 5-, 6-, and 7-positions of the heterocycle were evaluated (Table 1). Surprisingly, the evaluation of 5-FI revealed an unusual behavior: the inhibitory activity on the growth of the H37Rv Mtb strain was observed throughout the entire 96-well plate used in the assay (Supporting Information, Figure S1). This demonstrated that 5-FI can inhibit the growth of the bacillus with a high potency under the experimental conditions employed. The minimum inhibitory concentration (MIC) observed using three independent assays was 4.7 μM (0.63 μg/mL), although this value may have been underestimated due to the spreading of activity over the analysis plate, which may have reduced the concentration of the compound in the wells containing the highest molecule dilutions. One speculation for the observed behavior is the formation of a chemical structure of increased volatility, which could be responsible for the activity observed. However, the volatility of 5-FI itself is unlikely to be the cause as its melting and boiling points are 44.8 and 258 °C, respectively (ChemSpider, 2023). Another possibility is the enzymatic conversion of 5-FI to a compound with a higher vapor pressure. Enzymatic conversion of 5-FI to 5-FTrp is unlikely as the latter has a practically zero vapor pressure and a boiling point of 450.7 °C (ChemSpider, 2023). Further studies are thus needed to understand the unusual and somewhat puzzling data for 5-FI under the test conditions used to determine its MIC against the growth of the Mtb H37Rv strain.

**Table 1:**
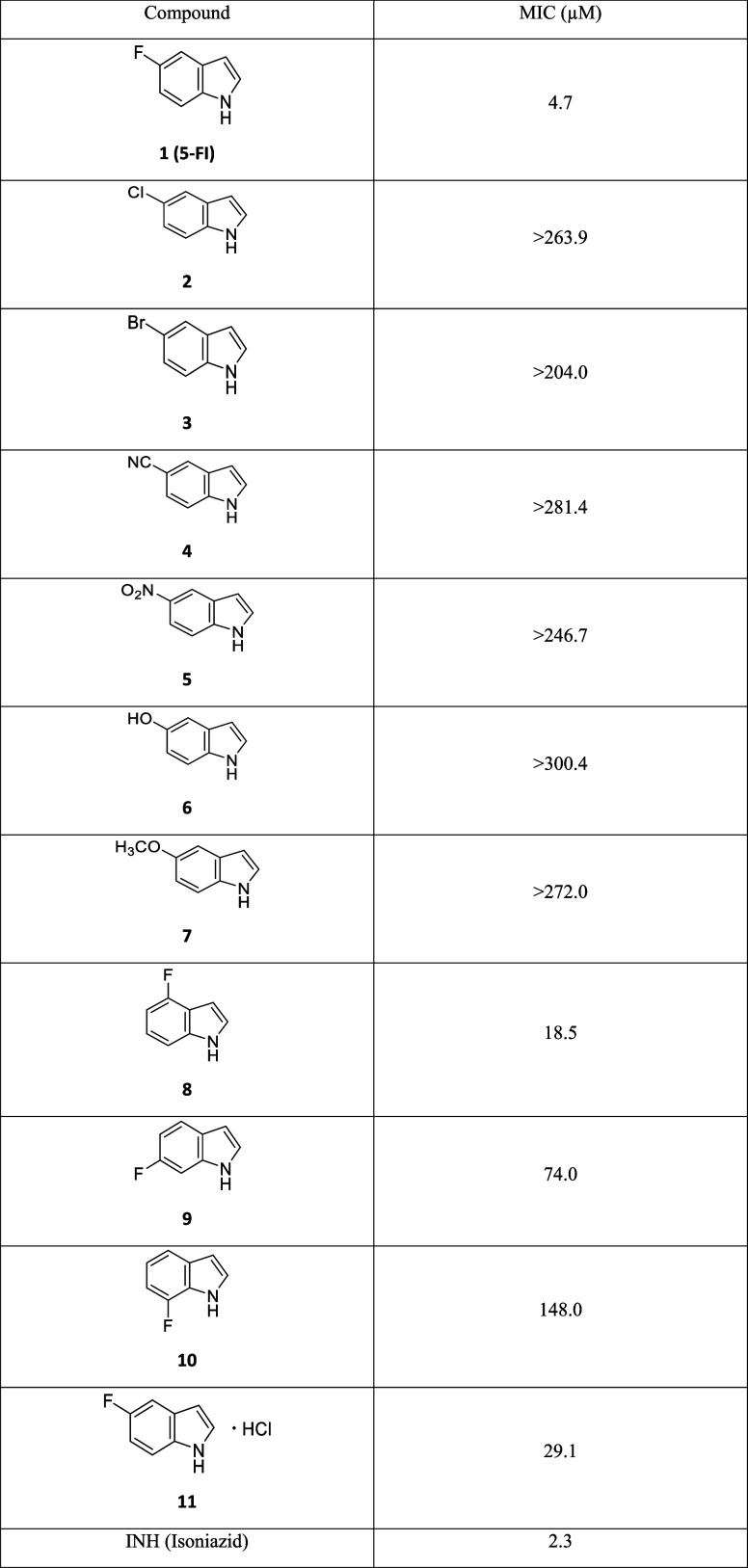
MIC of Compounds against the *Mycobacterium tuberculosis* H37Rv Strain

Indole derivatives containing electron-withdrawing (Cl, Br, CN, NO_2_) or electron-donating substituents (OH, OCH_3_) attached to the 5-position of the heterocycle were also evaluated against the Mtb H37Rv strain (Table 1). Interestingly, none of these substituents were able to maintain the antimycobacterial activity exerted by 5-FI, and the compounds were rather inactive at the highest concentrations tested. A comparison between 5-FI (**1**, MIC = 4.7 μM), 5-chloroindole (**2**, MIC > 263.9 μM), and 5-bromoindole (**3**, MIC > 204.0 μM) suggests that the increase in the atomic volume and polarizability of the halogen atom is detrimental to the capability of the chemical compound to arrest bacterial growth. These results suggest that steric hindrance may play a role in the ligand binding to the intracellular target. As qualitatively, polarizability measures how easily the electron density in an atom or a molecule can be perturbed by an external electric field (the larger and more diffuse the electron charge cloud, the greater its polarizability), the decreased polarizability of fluorine suggests that lower induced dipole moment in the molecule is favorable to ligand binding. Owing to the high electronegativity and low polarizability of fluorine, invoking σ hole involvement in ligand binding is not warranted. However, these conclusions should be considered with caution as they are based on MIC measurements and not target engagement (which is not known yet). The evaluation of the relationship between the position of the fluorine atom bound in the indole nucleus and the antimycobacterial activity demonstrated that its displacement to 4-, 6-, or 7-positions significantly reduces the inhibitory capacity of the compounds. Although the 4-FI MIC value of 18.5 μM was lower than the other 5-FI analogues tested, it was approximately 4-fold less active than 5-FI. Furthermore, 6-FI and 7-FI exhibited MICs of 74.0 and 148.0 μM, respectively. These data showed that shifting the fluorine atom from the 5-position of the indole nucleus in 5-FI to the 6- and 7-positions reduced the activity of the molecules by more than 15- and 31-folds, respectively.

Subsequently, 5-FI was assessed against a panel of Mtb-resistant strains obtained from clinical isolates (Table 2). Notably, the genomes of these drug-resistant strains were fully sequenced, and the genotypic changes responsible for resistance phenotypes are known.^24^*M. tuberculosis* PT2, PT12, and PT20 strains have been shown to be resistant to INH, rifampin, streptomycin, ethionamide, and rifabutine.^24^ The PT12 and PT20 strains are also resistant to pyrazinamide and ethambutol, and PT12 presents additional resistance to amikacin and capreomycin.^24^ 5-FI maintained an MIC value of 4.7 μM for pan-sensitive and drug-resistant strains that were evaluated (Table 2). These findings suggest that the 5-FI chemical compound exhibits promising activity against drug-resistant TB strains and does not share resistance mechanisms with the main clinically available drugs, likely acting through different biochemical pathways.

**Table 2:** Antimycobacterial Activity of 5-FI against Pan-Sensitive H37Rv and Drug-Resistant Strains of Mtb^a^

compound	MIC H37Rv (μM)	MIC PT2 (μM)	MIC PT12 (μM)	MIC PT20 (μM)	CC_50_^b^^,^^c^ HepG2 (μM)	CC_50_^b^^,^^c^ Vero (μM)
5-FI	4.7	4.7	4.7	4.7	>20	>20
INH	2.3	291.7	145.8	291.7		
RIF	0.09	>48.6	>48.6	>48.6		

a Viability of HepG2 and Vero strains in the presence of 5-FI after 72 h of exposure.

b Concentration required to reduce cell viability by 50% (CC_50_).

c Colorimetric assay based on 3-(4,5-dimethylthiazol-2-yl)-2,5-diphenyltetrazolium bromide conversion and neutral red incorporation assay. INH, isoniazid. RIF, rifampin.

To assess the selectivity and potential toxicity of 5-FI, the effects on the viability of both HepG2 and Vero mammalian cell lines were evaluated. Cell viability was measured after 72 h of incubation with concentrations of 2, 5, and 20 μM of the compound. The results demonstrated that the viability of the cell lines was not altered at any of these concentrations (Table 2). Two methods were used to assess the cell integrity: the MTT assay, which evaluates mitochondrial viability, and the neutral red assay, which assesses lysosomal functionality. These findings suggest that 5-FI can selectively inhibit Mtb without affecting mammalian cells’ viability at the tested concentrations, thereby indicating low cytotoxicity. Importantly, previous research has shown that 5-FI does not exhibit genotoxicity when evaluated using the SOS Chromotest.^25^ Interestingly, 4-FI, a 5-FI analogue, was found to be genotoxic using the same experimental conditions.

To determine the characteristics of 5-FI and the feasibility of employing the molecule in in vivo assays, the solubility of the compound was evaluated in different hydrogenionic contexts (Table 3). The purpose was to determine whether the compound was soluble at high concentrations that could allow its absorption at the observed pHs in the different compartments of the organism. The solubility in acidic pH (simulating the stomach pH) led to a higher solubility of the molecule (Table 3). At pHs 7.4 and 9.1, the solubilities presented were rather low (Table 3), being below its MIC value of 4.7 μM (Table 1), which could impair the pharmacokinetic exposure of 5-FI. Another parameter related to the pharmacokinetics of the compound was evaluated in the presence of rat liver microsomes. Data on the metabolic stability of the molecule demonstrated that the structure has an intrinsic clearance (Cl_int_) of 9.0 mL/min/kg and a half-life of 144.2 min (Table 3). These data suggest low hepatic metabolization of 5-FI, which could lead to a high pharmacokinetic exposure. In addition, the in vitro permeability was evaluated as a parameter indicative of 5-FI pharmacokinetic exposure in in vivo models (Table 3). The permeability of the compound using the PAMPA assay (parallel artificial membrane permeability assay) was 2.4.10^–6^ cm/s, indicating high permeability of the molecule, which bodes well for intestinal absorption and oral bioavailability.

**Table 3:** Solubility at Different pHs, Metabolic Stability, and Permeability of 5-FI

compound	aqueous solubility^a^			metabolic stability		permeability
	pH 1.2^b^ (mM)	pH 7.4^c^ (μM)	pH 9.1^d^ (μM)	Cl_int_^e^ (mL/min/kg)	*t*_1/2_^f^ (min)	PAMPA (10^–6^ cm/s)
5-FI	>550	3.5	2.2	9.0	144.2	2.4

a Solubility determined after incubation at 25 °C for 4 h. 0.1 M.

b HCl.

c PBS.

d 0.1 M NH_4_HCO_3_.

e Intrinsic clearance in the presence of rat liver microsomes.

f Half-life.

It was deemed appropriate to evaluate the chemical stability of 5-FI in aqueous solution to provide a solid basis on which to justify further efforts (Table 4). The molecule was evaluated using an open and closed system to assess whether the presence of air could catalyze any type of chemical degradation. In addition, the open system could provide an indication of a possible evaporation of the structure along with water molecules at 37 °C. First, an analytical method by HPLC was developed and validated to enable the quantification of 5-FI in solution. Evaluated for a period of 48 h, the compound showed limited stability under the test conditions used, with reduced recovery of the molecule as a function of time. It is important to highlight that the presence of air does not seem to significantly affect the stability of the structure since the concentrations were similar for the open and closed systems over 48 h of data collection (Table 4). Furthermore, the similar concentrations between the two experimental conditions allowed us to infer that the evaporation of 5-FI does not seem to be significant enough to drastically change the concentrations of the compound in solution at 37 °C.

**Table 4:** Chemical Stability of 5-FI in H_2_O at 37 °C

time (h)	open system (%)	closed system (%)
0	100 ± 0.0	100 ± 0.0
1	97.5 ± 0.2	97.5 ± 0.1
2	95.4 ± 0.2	95.2 ± 0.0
4	83.4 ± 0.7	93.3 ± 0.1
6	88.9 ± 0.2	87.8 ± 0.0
24	89.1 ± 0.2	86.1 ± 0.0
48	85.5 ± 0.1	80.3 ± 0.1

**Scheme 2:**
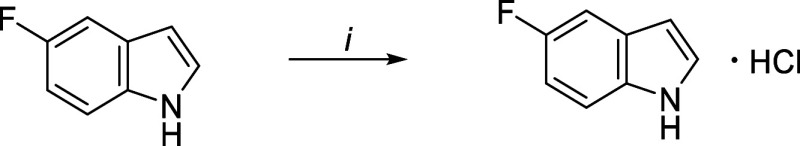
Conditions: *i* = H_2_O, HCl (6 M), 25 °C, 2 h

Thus, considering the low aqueous solubility at plasmatic and intestinal pH values of, respectively, 7.4 and 9.1, and the limited stability of 5-FI at 37 °C, the formation of salt of the compound was attempted. The alteration of the crystalline packing of the molecule by the formation of an ionic aggregate could improve the solubility and chemical stability of the structure. The first possible counterion evaluated was chlorine. 5-FI (1g) was dissolved in water (10 mL) and placed in the presence of HCl (≈6 M; 10–100 mL) at room temperature (25 °C) for 2h (Scheme 2). Solvent removal by lyophilization for 5 days yielded 90% of the hydrochloride.

The evaluation of the melting point of the formed product demonstrated that there was an increase in the temperature necessary to pass the compound from the solid to liquid state. The melting point of 5-fluoroindole hydrochloride (5-FI.HCl) was 124.2 °C, while 5-FI, as already mentioned, has a melting point of 44.8 °C. Furthermore, spectroscopic analyses using Fourier transform infrared (FTIR) confirmed the formation of 5-FI.HCl (Supporting Information, Figure S2).

As expected, the aqueous solubility of 5-FI.HCl was significantly increased at pH 7.4 and 9.1 (Table 5). The solubility of the hydrochloride was more than 171 times and 504 times greater than the solubility demonstrated by the free base (5-FI) at pH values of 7.4 (3.5 μM) and 9.1 (2.2 μM) (Table 3). On the other hand, in an acidic solution, the hydrochloride showed reduced solubility (2.3 mM) when compared to its free base (>550 mM). This behavior may be related to the common ion effect.

**Table 5:** Solubility at Different pH Values of 5-FI.HCl

compound	aqueous solubility^a^		
	pH 1.2^b^ (mM)	pH 7.4^c^(μM)	pH 9.1^d^(μM)
5-FI.HCl	2.3	600.2	1109.0

a Solubility determined after incubation at 25 °C for 4 h. 0.1 M.

b HCl.

c PBS.

d 0.1 M NH_4_HCO_3_.

The evaluation of stability and possible salt evaporation of 5-FI.HCl was measured in water at 37 °C using an open air and closed system during the time of the experiment (Table 6). Both the open system and the closed system showed no reduction in the concentration of the compound. By contrast, there was an increase in the concentration over time, probably related to the evaporation of water at 37 °C and the concomitant increase in the concentration of the resulting solution. Between the two evaluated systems, the use of a closed flask does not seem to prevent water evaporation. As observed, in 48 h, the mass concentration of 5-FI.HCl in the closed system was higher than the concentration in the open system. It should be pointed out that the recovery of 5-FI (Table 4) may be even lower over time as the evaporation of water in the experiment must have increased the final concentrations of the compound.

**Table 6:** Chemical Stability of 5-FI.HCl in H_2_O at 37 °C

time (h)	open system (%)	closed system (%)
0	100 ± 0.0	100 ± 0.0
1	97.6 ± 0.0	95.3 ± 0.1
2	98.0 ± 0.1	95.0 ± 0.2
4	100.3 ± 0.5	104.6 ± 0.2
6	103.9 ± 0.1	97.9 ± 0.1
24	113.5 ± 0.1	110.8 ± 0.2
48	114.3 ± 0.0	124.1 ± 0.1

The aqueous stability of 5-FI and 5-FI.HCl was performed by using different pH values that mimic different biological compartments (Table 7). These results show that 5-FI in the salt form shows increased aqueous stability at pH values 1.2 and 7.4. (Table 7). By contrast, there was a reduction of approximately 8.5% in the recuperation of 5-FI.HCl in comparison to that of 5-FI at pH 9.1 (Table 7).

**Table 7:** Chemical Stabilities of 5-FI and 5-FI.HCl^a^

chemical Stability			
compound	pH 1.2^b^ (%)	pH 7.4^c^(%)	pH 9.1^d^(%)
5-FI	37.2	58.5	99.2
5-FI.HCl	100	80.3	90.5

a Aqueous stability determined after incubation at 37 °C for 24 h. 0.1 M.

b HCl.

c PBS.

d 0.1 M NH_4_HCO_3_

Interestingly, although the 5-FI.HCl compound showed increased solubility at pH values of 7.4 and 9.1 (Table 5) and increased stability in aqueous solutions (Tables 6 and 7), data for intrinsic clearance (Cl_int_ = 48 mL/min/kg) and half-life (*t*_1/2_ = 12 min) showed decreased metabolic stability. These data suggest higher hepatic metabolization of 5-FI.HCl as compared to 5-FI, which could lead to lower pharmacokinetic exposure. The 5-FI.HCl chemical compound was further evaluated for its ability to be absorbed and reach the plasma after oral administration to mice (gavage). A comparison of the plasmatic concentrations observed between the hydrochloride (5-FI.HCl) and its free base (5-FI) was also made. The 5-FI compound was administered using a 4:1 mixture of propylene glycol (PPG):Tween-80, while the 5-FI.HCl was directly solubilized in 0.9% saline (0.9% NaCl). It should be pointed out that the difference between the vehicles used was due to the solubility of the compounds. After 15 and 30 min from administration, the animals were euthanized, and blood was collected for quantification of molecules in the plasma. An analytical method was developed and employed HPLC to detect either 5-FI or 5-FI.HCl chemical compounds. Administration by gavage of 400 mg/kg of either 5-FI or 5-FI.HCl provided plasma concentrations that ranged from 4.7 to 18.1 μg/mL (32.6–82.3 μM) (Table 8). The plasma concentration values were 9.6- up to 16.9-fold larger than the amount of 5-FI needed to inhibit Mtb growth in vitro (MIC = 4.7 μM), whereas these values were between 1.1- and 2.8-fold larger for 5-FI.HCl (MIC = 29.1 μM). The data obtained in the experiment did not allow inference of significant differences in the absorption of the structure in the form of free base or hydrochloride. Furthermore, plasma concentrations were time-dependent, with the highest values reached after 30 min following oral administration. The results of this evaluation allow us to conclude that the compounds are absorbed after administration by gavage in mice and reach plasmatic concentrations above the MIC after 15 min.

**Table 8:** Plasma Concentrations of 5-FI and 5-FI.HCl after Gavage Administration to Mice

compound[] = 400 mg/kg	time (min)	mean ± SD (μM)
5-FI	15	45.2 ± 14.8
5-FI	30	79.4 ± 19.4
5-FI.HCl	15	32.6 ± 14.7
5-FI.HCl	30	82.3 ± 35.05

As besides increased solubility and chemical stability, 5-FI.HCl showed both good absorption and ability to reach the systemic circulation of animals without the need to use vehicles containing cosolvents or surfactants, this compound was chosen to evaluate its effectiveness in the model of TB in mice. However, before embarking on in vivo studies, the structure was evaluated for the ability to inhibit the growth of the Mtb H37Rv strain in vitro. The MIC for 5-FI.HCl (29.1 μM) increased compared to the MIC obtained for 5-FI (4.7 μM) under the same experimental conditions (Table 1). This result is likely related to the greater polarity of the hydrochloride, which may have resulted in the reduced ability to cross the mycobacterial cell wall. It is important to highlight that the activity spreading pattern observed in the test using 5-FI (Supporting Information, Figure S1) was significantly reduced but not totally eliminated.

**Figure 2:**
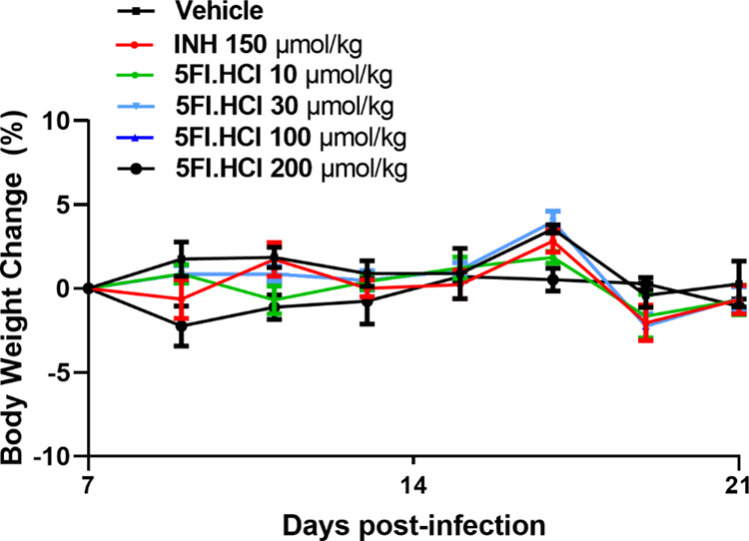
Variation in the animals’ weight during the evaluation of the effectiveness of 5-FI.HCl in the TB model in mice.

To evaluate the effectiveness of 5-FI.HCl on Mtb in vivo infection, groups of animals were randomized and infected with 10^6^ viable cells of the H37Rv strain. After new randomization, the animals were divided into the following groups: early control (EC), INH (150 μmol/kg), vehicle (NaCl 0.9%), and groups treated with 5-FI.HCl (10, 30, 100, and 200 μmol/kg). The EC group of animals was used to evaluate the initial mycobacterial load of the experiment and thus did not receive any type of treatment. The EC group was euthanized on the seventh day after infection. For the other groups, on the seventh day after infection, treatments were initiated using a single daily dose administered by gavage. Lung homogenates were separately plated and incubated for 21 days (37 °C) to count the number of viable Mtb colonies. In addition, the spleens of the animals were weighed to assess whether any inflammatory process was developed after Mtb infection. It is important to point out that after a period of 15 days of treatment, no deviation in the behavioral and clinical profile was observed for all animals belonging to this study. The absence of change in body mass during treatment can be considered an indication of the low toxicity of the evaluated compounds (Figure 2). The animals’ weight was evaluated every 2 days during the treatment period.

After 15 administrations (days 7–21), all groups were euthanized, and the samples were processed accordingly. The concentrations of 10, 30, and 100 μmol/kg of 5-FI.HCl were not able to reduce the number of colony forming units (CFUs) in the lungs of the animals when compared to the group treated with vehicle (Table 9). In addition, the animals’ spleen mass also remained fairly similar at these concentrations of 5-FI.HCl when compared to the group that received only saline solution (Table 9). These data indicated the maintenance of the inflammatory process in this primary lymphoid organ even after treatment with 5-FI.HCl at concentrations of 10, 30, and 100 μmol/kg. On the other hand, the administration of 200 μmol/kg of 5-FI.HCl was able to significantly reduce the mycobacterial load in the lungs of the animals (Table 9). The spleen mean mass of mice treated with 200 μmol/kg 5-FI.HCl showed a tendency (*P* = 0.67) to decrease compared to the group that received only the administration vehicle. The same trend was observed for INH (*P* = 0.48), a first-line drug used for the treatment of tuberculosis, which also showed a reduction in the spleen mass in the group of animals treated with 150 μmol/kg of the drug. Interestingly, as observed for INH, the administration of 200 μmol/kg of 5-FI.HCl significantly reduced the number of CFUs when compared to the EC group (Table 9), suggesting that this concentration had a bactericidal effect on the bacillus present in the lungs of animals. As the early control group represents the initial mycobacterial load (T0) in the lungs, the reduction of CFU from that point onward indicates that the compound not only limited the infection (bacteriostatic effect) but was able to reduce the viability of the bacillus to levels lower than that of the beginning of treatment (bactericidal effect).

**Table 9:** Counting of CFUs and Determination of Spleen Mass in Animals in a Model of Tuberculosis in Mice^a^

groups	lungs	spleen weight
	mean log_10_ CFU/mL ± SD	mean g ± SD
5-FI.HCl (10 μmol/kg)	5.80 ± 0.20	0.58 ± 0.19
5-FI.HCl (30 μmol/kg)	6.21 ± 0.32	0.55 ± 0.23
5-FI.HCl (100 μmol/kg)	5.86 ± 0.67	0.62 ± 0.35
5-FI.HCl (200 μmol/kg)	3.68 ± 0.45***^, ###^	0.28 ± 0.06
INH (150 μmol/kg)	2.43 ± 0.40***^, ###^	0.23 ± 0.04
vehicle	5.62 ± 0.60	0.49 ± 0.11
EC	4.85 ± 0.32	0.34 ± 0.09

a Data represent mean ± standard deviation. Statistical analysis was performed by one-way ANOVA followed by Tukey’s multiple comparisons test using GraphPad Prism 9.0.0 software. ***P < 0.001 compared to vehicle (0.9% NaCl) group. ^###^*P* < 0.001 compared to the EC group. *N* = 4–10 animals per group. 5-FI.HCl, 5-fluoroindole hydrochloride. INH, isoniazid. EC, early control.

## Conclusions

Herein, we have shown that 5-FI in the free base and hydrochloride salt forms is an inhibitor of cell growth of the *M. tuberculosis* pan-sensitive H37Rv strain and drug-resistant clinical isolates. Indole is an intermediate of the six-step biosynthesis of tryptophan, and the addition of an intermediate may represent a lower energetic cost for the synthesis of this often limiting amino acid for protein biosynthesis.^26^ Metabolic tracing studies showed that 5-fluoroanthranilate is rapidly converted to 5-fluorotryptophan that accumulates in the bacilli.^21^ It has also been shown that 4-fluorotryptophan is incorporated into the cytosolic proteins of the *M. tuberculosis* mc^2^ 6230 strain.^21^ Moreover, tryptophan acts as an allosteric inhibitor of anthranilate synthase.^21^ It was thus deemed justifiable to test 5-FI as a potential inhibitor of mycobacterial cell growth. Accordingly, it is here proposed that 5-FI may act as a cosubstrate joined with l-serine to C3 for the PLP-dependent β-subunit of bifunctional tetrameric tryptophan synthase (α_2_β_2_), yielding 5-fluorotryptophan. No other substituent in the 5-position indole ring, electron donors (OH, OCH_3_) and withdrawing groups (Cl, Br, CN, and NO_2_), resulted in the same level of inhibition of Mtb growth. The position of fluoro substituent in the indole core proved to modulate the Mtb inhibition activity of the compounds. The fluorine atom in the indole ring 4-, 6-, and 7-positions did not reach the same potency of 5-FI. Importantly, 5-FI exhibited low cytotoxicity against HepG2 and Vero lineages in the concentrations of 2, 5, and 20 μM, indicative of selectivity. The compound was found to be permeable using the PAMPA assay (2.4.10^–6^ cm/s) and was associated with high metabolic stability with an intrinsic clearance of 9.0 mL/min/kg in rat liver microsomes. It is worth mentioning that 5-FI remained stable >50% at pH 7.4 and >99% at pH 9.1. 5-FI.HCl, the hydrochloride salt of 5-FI, was >80% stable at all pH values tested. The in vivo inhibitory activity was evaluated in CF-1 mice. In these experiments, both 5-FI and 5-FI.HCl were quantified in plasma, with concentrations ranging from 79 to 82 μM after 30 min of administration of an oral dosage of 400 mg/kg/animal. The model of infection showed reduction of bacterial burden in the concentration of 200 μmol/kg 5-FI.HCL (more available in plasma) with no evidence of a toxic effect up to 21 days of treatment. The results here described show that 5-FI.HCl exhibits in vivo bactericidal activity. No inhibition of Mtb H37Rv strain growth was observed for 5-fluorotryptophan at concentrations up to 20 μg/mL (approximately 90 μM) under the same experimental conditions employed by the other indole-containing chemical compounds presented in Table 1. This lack of growth inhibition may be ascribed to both the rather large Michaelis–Menten constant (*K*_M_ = 1.25 mM) for the tryptophan transport system of Mtb H37Rv strain^27^ and the increased polarity of 5-fluorotryptophan (reduced passive diffusion). The results for 5-FI.HCl suggest that this compound may be a promising drug candidate for treating TB with the potential to inhibit drug-susceptible and drug-resistant Mtb strains. Even though for the latter strains further efforts are needed, the results here described indicate that these studies are warranted and ought to be pursued in the near future. However, the results presented here do not provide any experimental evidence that 5-fluorotryptophan has been biosynthesized. Accordingly, analysis of mass spectrometry data (e.g., LC-TOF-MS) of the metabolic profile of *M. tuberculosis* after in vitro treatment with 5-FI (or 5FI.HCl) should be pursued to provide a solid basis to shown whether or not it has been incorporated into polypeptide chains. Moreover, the impact of incorporating 5-fluorotryptophan into, for instance, enzymes must be based on its involvement in catalysis and/or substrate binding via π interactions as fluorine is a strong electron-withdrawing group. Finally, it has been pointed out that further validation of tryptophan biosynthesis as a drug target in mycobacteria will require genetic and infection treatment experiments owing to the diversity and complexity of microenvironments in which the bacilli reside.^16,21^

## Materials and Methods

### Chemistry

Reactants and solvents were obtained from commercial suppliers and were used without additional purification. The melting points were measured using a Microqumica MQAPF-302 apparatus. FTIR spectra were recorded using a universal attenuated total reflectance attachment on a PerkinElmer Spectrum 100 spectrometer in the wavenumber range of 650–4000 cm^–1^ with a resolution of 4 cm^–1^.

### MIC Assay

The MIC was conducted in 96-well plates using the REMA, as previously described.^28^ Resazurin (7-Hydroxy-3H-phenoxazin-3-one 10-oxide; blue/purple color) is reduced to resorufin (7-Hydroxy-3H-phenoxazin-3-one; pink color) by aerobic respiration of metabolically active cells and is, therefore, used as an indicator of cell viability. Indoles were solubilized in 100% DMSO (Sigma-Aldrich, St. Louis, MO, USA) to a concentration of 2 mg/mL and stored at −20 °C until the day to be tested. After room temperature was achieved, indoles were diluted in Middlebrook liquid medium (Becton Dickinson, BD) containing 10% (v/v) BBTLM Middlebrook ADC enrichment (albumin, dextrose, and catalase, BD) and 5% (v/v) DMSO concentrations limited by the compound solubility. The Mtb H37Rv laboratory reference strain (ATCC 27294) was used to evaluate the biological activity over both pan-sensitive strain and three drug-resistant clinical isolates of Mtb (PT2, PT12, and PT20), isolated from patients in the Lisbon Health region, Lisbon, Portugal.^24^ INH (Acros) was used as a positive control. The compounds to be tested were 2-fold serially diluted, resulting in a concentration range that variated 10-point. The active compounds prevented the reduction of resazurin, observed in the well plate, marked by the change of color–from blue to bright pink–and the MIC result considered the lowest compound concentration capable of preventing this change of color and the respective Mtb growth. All assays were performed in triplicate for each compound on different days, and the result, reported in μM unit, was the most frequent value achieved.

### 5-FI Salt Preparation

To convert the 5-FI into its hydrochloride presentation, 1g of commercial 5-FI was weighed into a 25 mL reaction flask and reacted with 10 mL of ∼6 M hydrocloric acid with strong stirring at RT for 2 h. Afterward, the mixture was frozen and liofilized for 5 days. The obtained product was used without an additional purification.

### HPLC Analysis

The experiments were carried out in two pieces of equipment. They were equipped with a DA detector, autosampler, column thermostat, and degasser. To analyze 5-FI, a Dionex ultimate 300 and also a Shimadzu HPLC both with a C18 5 μm Nucleodur column (250 × 4.6 mm) were used. A method was constructed with 1% acidic acetic as the mobile aqueous phase. The mobile phase used to compose the gradient was acetonitrile:methanol (1:1) in the gradient mode: at 0 min 100% 1% acetic acid, at 6 min 10% acetic acid, 1 and 90% acetonitrile:methanol (1:1), at 10 min the content was back at 100% 1% acetic acid 1% and the re-equilibration was performed for more 6 min until the end of the program. The column oven temperature was 30 °C, and the flow was set to 1.5 mL/min. The calibration curves were constructed in the following concentrations: 0.25, 0.5, 1.0, 5.0, 10.0, and 50 μg/mL in triplicate.

### Analytical Curve

All of the curves were constructed starting from a 1 mg/mL stock solution prepared into the solvent used to construct the analytical curve. To obtain the analytical curves used in the volatility assay, the calibration curves were constructed in the following concentrations: 0.25, 0.5, 1.0, 5.0, 10.0, and 50 μg/mL, in triplicate separately for water and 7H9 medium, with the same HPCL method described above. For the plasma analysis, the concentrations were 0.125, 0.25, 0.5, 7.5, and 10 μg/mL.

### Volatility Assay

The compound was prepared in a 1.5 mL microtube with a final volume solution of 1 mL in ultrapure water or 7H9 medium at 10 μM concentration and incubated at 37 °C for 7 days. In the first day, aliquots were taken hourly from 1 to 6 h of incubation and then by day until the seventh day, which represents the time of incubation of minimum inhibitory Mtb assay. The assay was conducted in two ways: with the microtube open and closed for water or 7H9 as the solvent. All aliquots were centrifuged at 17.000 × *g* for 20 min, and the supernatant was filtered through a 0.22 μm PDVA filter. The samples were analyzed by HPLC, and their areas were plotted on the previously constructed analytical curve.

### Cellular Viability

To determine the cellular viability, the cells were incubated with the 5-fluoro-*1H*-indole compound with 3-(4,5-dimethylthiazol-2-yl)-2,5-diphenyl-2H-tetrazolium bromide (MTT)^29^ and neutral red uptake (NRU)^30^ methods. In the Dulbecco’s modified medium Eagle Medium (DMEM-Invitrogen, Waltham, MA, USA), HepG2 and Vero cells were grown supplemented with 10% inactivated fetal bovine serum (Invitrogen), 1% penicillin-streptomycin (Invitrogen), and 0.1% fungizone (Invitrogen). In a 96-well culture plate, HepG2 (4 × 10^3^ cells/well) and Vero cells (2 × 10^3^ cells/well) were seeded and incubated for 24 h. The compound was diluted in three concentrations, 2, 5, and 20 μM, using 5% DMSO and were incubated with the cell lines for 72 h at 37 °C. In the MTT assay, after this period of incubation under 5% of CO_2_, the cultures were mixed with the MTT solution (5 mg/mL) for 4 h. The formed formazan crystals were dissolved with 100 μL of DMSO, and the absorbance of this solution was measured at 595 nm using an EZ Read 400 microplate reader (Biochrom, Holliston, MA, USA). The absorbance of the formazan crystals, diluted with DMSO, was directly proportional to the number of live cells with active mitochondria, corresponding to their viability. The mean value of absorbance of control wells was taken as 100% viability, and the values of treated cells were calculated as the percentage of vehicle control containing 0.5% DMSO. In the NRU assay, cells were incubated for 72 h and washed with PBS solution, and 200 μL of neutral red dye solution (25 μg/mL, Sigma) prepared in serum-free medium was added to the plate and incubated for 3 h at 37 °C under 5% of CO_2_, and 100 μL of desorbing solution (ethanol/acetic acid/water (50:1:49) was added followed by gentle shaking for 30 min after washing with PBS. The absorbance of neutral red dye extracted from the viable cells in the process was measured at 562 nm using an EZ Read 400 microplate reader (Biochrom, Holliston, MA, USA). Considering the vehicle control cell (0.5% DMSO) as 100% cell viability, the results were expressed in the percentage of viable cells.

### Parallel Artificial Membrane Permeability Assay

5-FI was quantified by HPLC-MS after a period of incubation and separated into two fractions by a permeable lipidic membrane. The result is expressed in velocity units of permeation (cm/s). The lipidic membrane is previously prepared with specific activation solutions that create a hydrophobic surface, simulating the intestinal epithelial tissue. To the donor aqueous phase (pH 7.4), 100 μM compound is added. To the previously prepared membrane is added the receptor aqueous phase (pH 7.4), and the mixture is incubated for 5h at room temperature with both receptor and donor aqueous phases in contact, separated just by the membrane. The compound transported by passive diffusion is measured by taking an aliquot of receptor solution and analyzing it by HPLC-MS/MS.

### Metabolic Stability

The compound was incubated at 37 °C in the concentration of 20 μM with rat liver microsomal fractions in PBS solution at pH 7.4 solution. As previously described,^31^ rat liver microsomal fractions were seeded by centrifugation at 9.000 × *g*. To the supernatant containing the postmithocondrial with cytosolic and membrane-bound enzymes, 4 μM 5-FI was added and incubated at 37 °C. The interaction between the compound in the incubation mixture and the enzyme containing NADPH at 1 mg/mL concentration was measured by HPLC-MS/MS technique to determine the in vitro compound consumption by the microsomal fractions at 0, 5, 15, and 30 min reaction (incubation) time. The intrinsic compound clearance was taken as the half-life, and the disappearance was compared with the positive control, verapamil(2 μM). The values for have been described as low (<5 mL/min/kg), moderate (5–15 mL/min/kg), and high (>15 mL/min/kg) for intrinsic clearance.^32^

### Chemical Stability

The compounds to be tested were prepared in a 100 μM solution and incubated at 37 °C for 24 h in three solutions representing the physiologically relevant pHs to drug administration: pH 1.2 (stomach), pH 7.4 (plasma), and pH 9.2 (intestinal tissue). The compounds were quantified by HPLC-MS/MS. The chemical stability experiments were carried out in triplicates to ensure reproducibility. The results were presented as percentage, comparing the signal at time zero of the assay, considering this measure 100%, with measurements after the incubation time. Alprenolol was used as the analytical control (data not shown). The results were presented as the percentage, comparing the signal at time zero of the assay (100%) with the signal produced by concentrations after the incubation period.

### Solubility Assay

The compound was weighed (1 mg) and solubilized in phosphate-buffered saline (PBS, pH 7.4), HCl 0.1 M (pH 1.2), and NH_4_HCO_3_ 0.1 M (pH 9.2) to a final concentration of 1 mg/mL. The solutions were kept under stirring (250 rpm) for 4h at 37 °C. The solutions were filtered (0.22 uM PDVA filter) and analyzed by HPLC. DMSO solution 1 mg/mL was used as the control for 100% soluble.

### In Vivo Absorption Assay

Two different formulations were teste, using female CF-1 mice (*n* = 2). A single dose of 400 mg/kg, formulated in either 0.9% saline solution or propylene glycol:Tween-80 (4:1),^33^ was administered by gavage, and after 15 and 30 min, the animals were euthanized and the blood collected in a tube containing heparin (BD Vacutainer, USA) and kept on ice. The samples were centrifuged at 17.000 × *g* for 30 min, and the supernatant was collected. To 75 μL of supernatant containing the compound, 25 μL of trichloroacetic Acid (TCA), which is a deproteinizing agent, was added, and the plasma sample was centrifugated at 17.000 × *g* for 20 min. The supernatant was collected in a vial containing a restrictor, the sample was analyzed by HPLC, and the data were plotted on the analytical curve. The methodology applied to the assay was approved by the Animal Ethics Committee from Pontifcia Universidade Católica do Rio Grande do Sul (CEUA/PUCRS) (Protocol *N*°: SIPESQ/CEUA: 10649).

### In Vivo Model of TB Infection

The evaluation of pharmacological activity of 5-Fluoro-1H-indole hydrochloride was performed in accordance with a previously reported methodology,^34^ with some modifications. One isolated colony of the laboratorial H37Rv strain was cultured in 5 mL of 7H9 medium supplemented with ADC, 0.05% (v/v) Tween-80, and 0.02% (v/v) of glycerol until reaching the mid log phase. The animals were maintained under the following conditions: 12/12h dark/light cycle, food and water *ad libitum*, 40–60% humidity, and 24 ± 2 °C. Mice were anesthetized by intraperitoneal injection of ketamine (100 mg/kg) and xylazine (10 mg/kg) mixture. Then, the suspension of 10^6^ viable H37Rv strain cells in 200 μL of saline solution was administered through the retroorbital venous plexus. The treatment was continued for 7 days after the beginning of infection. The EC group was euthanized soon after infection to allow assessment of whether or not there was any bactericidal effect of the compound on mycobacterial growth. The groups were randomly separated into 5-FI 10, 30, 100, and 200 μmol/kg solution in saline. INH (150 μmol/kg) was used as the positive control, and saline solution was used as the negative control. The groups were treated daily by administering a single dose by gavage for the subsequent 15 days. To assess the lung and spleen CFU counts as well as splenomegaly, mice were euthanized by isofurane inhalation after 2 days of the end of treatment. First, the spleen was weighed, and subsequently lung and spleen were homogenized in a glass tissue homogenizer (Góes Vidros especiais, Porto Alegre -RS, Brazil) containing 3 mL of saline solution. To measure the number of viable organisms, the tissue homogenates were serially diluted into agar plates containing Middlebrook 7H10 (Difco, Sparks, MD) supplemented with 10% oleic acid–albumin–dextrose–catalase enrichment (Becton Dickinson, Franklin Lakes, NJ) and 0.2% (v/v) glycerol. Plates were incubated at 37 °C approximately 21 days prior to counting viable Mtb H37Rv cells. Finally, for *M. tuberculosis* cell counts, the numbers were converted into logarithms of CFU (log_10_ CFU). Data were evaluated by one-way analysis of variance (ANOVA) followed by Tukey’s post-test using GraphPad Prism 9.0 (GraphPad Software Inc., San Diego, CA). Significance between groups was determined using *P* < 0.05. Animals with a variation above of two standard deviations, compared within their own group, were considered outliers and were excluded from the analysis. Using this criterion, two animals were excluded from lung evaluation (EC and 150 μmol/kg groups) and one from spleen (EC group). All experimental protocols were approved by the Animal Ethics Committee from Pontifícia Universidade Católica do Rio Grande do Sul (CEUA-PUCRS) (protocol number:8637).

## Supporting Information Available

Spreading pattern in the REMA assay and FTIR spectra comparing 5-FI/5FI.HCL (PDF)
