# Supplementary material for: 5-Fluoroindole Reduces the Bacterial Burden in a Murine Model of Mycobacterium tuberculosis Infection
Source: ACS Omega. 2024 Jul 16;9(30):32969–79. doi: 10.1021/acsomega.4c03981 (PMC11292626; doi:10.1021/acsomega.4c03981)
Supplement: Supplementary file 1 — ao4c03981_si_001.pdf [file ao4c03981_si_001.pdf]

5-Fluoroindole reduces the bacterial burden in a murine model of *Mycobacterium tuberculosis* infection

Christiano E. Neves[a,b], Josiane D. Paz[a,b], Bruno L. Abbadi[a], Raoní S. Rambo[a], Alexia M. Czeczot[a,b], Nathalia D. M. Sperotto[a], Adilio S. Dadda[a], Rodrigo B. M. Silva[a], Marcia A. Perelló[a], Guilherme A. Gonçalves[a,c], Laura C. González[a,b], Cristiano V. Bizarro[a,b], Pablo Machado[a,b,c], Luiz A. Basso[a,b,c]**

[a]*Instituto Nacional de Ciência e Tecnologia em Tuberculose, Centro de Pesquisas em Biologia Molecular e Funcional, Pontifícia Universidade Católica do Rio Grande do Sul, 90616-900, Porto Alegre, Rio Grande do Sul, Brazil*

[b]*Programa de Pós-Graduação em Biologia Celular e Molecular, Pontifícia Universidade Católica do Rio Grande do Sul, 90616-900, Porto Alegre, Rio Grande do Sul, Brazil*

[c]*Programa de Pós-Graduação em Medicina e Ciências da Saúde, Pontifícia Universidade Católica do Rio Grande do Sul, 90616-900, Porto Alegre, Rio Grande do Sul, Brazil*

Table of contents

Spreading pattern in the REMA assay S1

FTIR spectra of synthesized compounds S2

Figure S1. Spreading pattern in the REMA assay

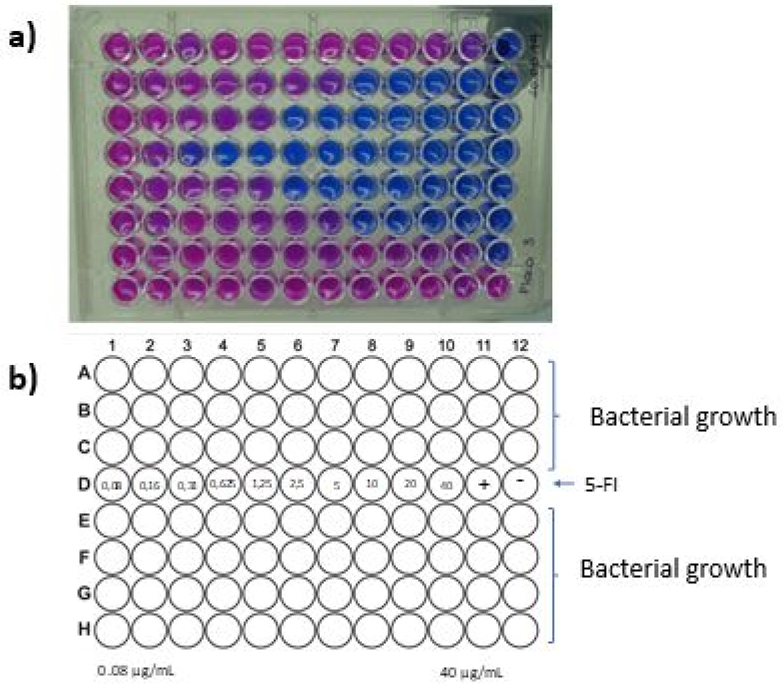

Figure S1: (a) 96-well plate representing one of the 3 independent experiments carried out to determine the MIC for 5-FI. (b) The schematic representation of the experiment, showing the evaluated dilutions. In the presence of resazurin, the color turns bluish in the absence of bacterial growth and pink when there are viable bacteria in the sample.

Figure S2. FTIR spectra comparing 5-FI/5FI.HCL

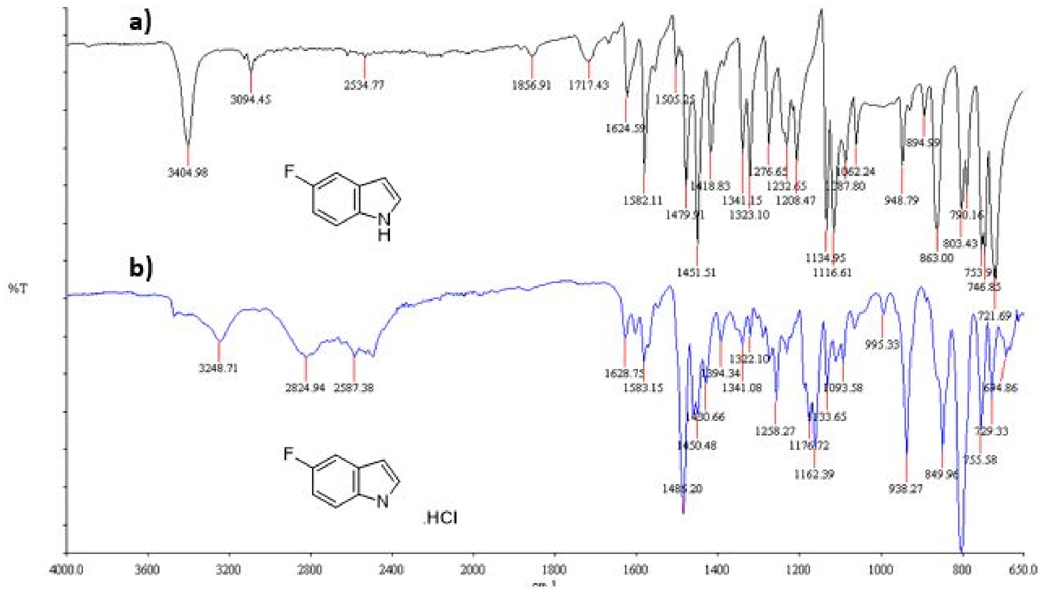

Figure S2: (a) 5-FI FTIR spectra. (b) 5-FI.HCl FTIR spectra.
